# The Relationship Between Quarantine Length and Negative Affect During the COVID-19 Epidemic Among the General Population in China: The Roles of Negative Cognition and Protective Factors

**DOI:** 10.3389/fpsyg.2021.575684

**Published:** 2021-04-28

**Authors:** Lulu Hou, Fangfang Long, Yao Meng, Xiaorong Cheng, Weiwei Zhang, Renlai Zhou

**Affiliations:** ^1^Department of Psychology, Shanghai Normal University, Shanghai, China; ^2^Department of Psychology, Nanjing University, Nanjing, China; ^3^School of Nursing, Nanjing Medical University, Nanjing, China; ^4^School of Psychology, Central China Normal University, Wuhan, China; ^5^Department of Psychology, University of California, Riverside, CA, United States

**Keywords:** COVID-19, quarantine, depression, anxiety, worry, anticipation, protective factor

## Abstract

Quarantine and isolation at extended length, although considered as highly effective countermeasures for the novel coronavirus (COVID-19) which started at the end of 2019, can have great impact on individual's mental health, especially emotional state. The present research recruited 5,115 participants from the general public across 32 provinces and autonomous regions in China in an online survey study, about 20 days after the lockdown of the epicenter (Wuhan), to investigate the relationship between the length of the quarantine and negative affect (including depression and anxiety), as well as the mediating roles of negative cognition (including worry and anticipation), and the moderating roles of dispositional optimism, tolerance of uncertainty, social support, and healthy behavior. The results showed that: (1) Worry and anticipation mediated the relationship between quarantine length and depression and anxiety; (2) Dispositional optimism moderated the path coefficients of quarantine length to worry, worry to anxiety, and anticipation to depression; (3) Tolerance of uncertainty moderated the path coefficient of worry to anxiety; (4) Social support moderated the path coefficient of anticipation to anxiety. In conclusion, during quarantine, dispositional optimism, uncertainty tolerance, and social support can buffer the direct or indirect effects of quarantine length on depression and anxiety. These findings could have profound implications on the societal responses to COVID-19 and future pandemics.

## Introduction

Due to the high infectivity of the novel coronavirus disease (COVID-19), Wuhan was locked down on January 23, 2020 to curb the further deterioration and spread of COVID-19. On January 30, 2020, the World Health Organization (WHO) declared the outbreak of COVID-19 as an international public health emergency. On March 11, 2020, the WHO further declared COVID-19 as a pandemic largely due to the uprising COVID-19 cases in Europe, especially Italy, and the United States. As of today (mid-May), confirmed cases in the United States reached 1.5 million.

As the first country to fight the COVID-19 epidemic, China has accumulated valuable experience in detection, clinical diagnosis and treatment, epidemiological statistics, and transmission control. The sharp decrease in the number of confirmed cases within two months in China after the Wuhan lockdown has demonstrated that self-quarantine/isolation^1^ play a role in controlling the spread of COVID-19. However, self-quarantine with extended duration can have various negative impacts on mental health in the individuals in isolation. Some recent studies have documented depression and anxiety of different degrees at various age groups (Cao et al., 2020; Wang et al., 2020a) that can stay for at least 4 weeks (Wang et al., 2020b) during the COVID-19 epidemic in China.

However, little empirical research has assessed the potential mediating processes on the effects of quarantine on depression and anxiety, even though this knowledge could be important for the general public to cope with the potential mental health issues from extended isolation. The present study was thus carried out about 20 days after the lockdown of Wuhan, the epicenter of the coronavirus outbreak in China. At that time, as the number of confirmed cases in China was still rising, many cities across the country were in self quarantine at home. We assessed various individual difference factors, including worry, anticipation, dispositional optimism, tolerance of uncertainty, social support, and healthy behaviors, for the relationship between quarantine length and negative affect.

According to Schulz and Lazarus (2012), various cognitive factors mediate the relationship between stimulus and emotion, and the resulting emotional experience (e.g., how people interpret or evaluate the emotional stimulus). One of the cognitive factors is worry, the thinking of a problem and the cognitive tendency that cannot be relieved (Mennin et al., 2004). Worry often shows a unique relationship with generalized anxiety disorder and is also common in a variety of mental disorders, including depression (Mohlman et al., 2004; Gladstone et al., 2005). Specifically, cancer patients' worry about losing life can affect their depression (Rao et al., 2017). Therefore, we hypothesized that the anticipation of infection and worry would mediate the relationship between quarantine length and depression and anxiety.

The extent to which the individuals are more less affected by quarantine and negative cognition (including worry and anticipation) may be closely related to the inner resources such as dispositional optimism. Dispositional optimism is defined as the belief that future events may have positive results (Lai and Yue, 2000; Jiang et al., 2016). It can provide psychological capital to support individual's growth and development with positive psychological state. Dispositional optimism, as an important predictor of subjective well-being (Scheier and Carver, 1992), is often associated with less pain in difficult times (Taylor et al., 1990). It is thus expected that individuals with high levels of dispositional optimism will show less stress, depression, and loneliness, but receive more social support, than those with low levels of dispositional optimism (Taylor et al., 1990). Dispositional optimism can further promote mental and physical health by buffering the impact of depressing events with positive emotions (Scheier and Carver, 1992). Therefore, we hypothesized that dispositional optimism would moderate the relationship between quarantine length and negative affect and the mediating effect of negative cognitions.

In addition, given the extreme uncertainty regarding the quarantine length and whether the individual will be infected during the COVID-19 epidemic, another important individual factor will be tolerance of uncertainty. It is the set of negative and positive psychological response—cognitive, emotional, and behavioral—provoked by the conscious awareness of the lack knowledge about particular aspects of the world (Hillen et al., 2017). Specifically, individuals who cannot tolerate uncertainty tend to treat ambiguity as a source for stress, frustration, and anxiety, and also to avoid uncertainty as much as possible. For these individuals, various daily events that involve different degrees of uncertainty could trigger negative emotional experiences. Accordingly, individuals with low tolerance of uncertainty tend to show higher levels of depression and anxiety (Dar et al., 2017). We thus hypothesized that tolerance of uncertainty would moderate the relationship between quarantine length and negative affect and the mediating effect of negative cognitions.

According to the buffer model of social support (Thoits, 1982), social support can play an important role for people faced with high-pressure situations by reduce the impact of negative emotions in the following aspects. Firstly, social support can influence people's subjective evaluation of society, which can make individuals feel less stressful in the face of pressure; Secondly, social support can buffer the negative impacts of diseases; Thirdly, social support can help problem solving at difficult times. It is thus expected that social support during quarantine will protect mental health. Accordingly, empirical research has shown that social support can reduce depression and anxiety in cancer patients (Kornblith et al., 2001), predict subsequent depressive symptoms (Khatib et al., 2013), moderated the relationship between stress and depression, anxiety (Raffaelli et al., 2013), and the relationship between acute stress and emotional symptoms (Guo et al., 2020). We thus hypothesized that social support would moderate the relationship between quarantine length and negative affect and the mediating effect of negative cognitions.

As for the last factor, there is a large literature demonstrating the effects of healthy behaviors on mental health. For instance, exercise and healthy diet can reduce depression and anxiety (Byrne and Byrne, 1993; Saneei et al., 2016; Trudel-Fitzgerald et al., 2016). It is therefore expected that healthy behavior habits during quarantine will improve the emotional state of individuals, and may subsequently break the vicious circle of “quarantine length—negative cognition—negative affect” (i.e., our hypothesis that anticipation and worry about getting infected mediate the relationship between quarantine length and negative affect). Specifically, we hypothesized that healthy behaviors would moderate the relationship between quarantine length and negative affect and the mediating effect of negative cognitions.

In summary, the present study assessed several individual difference factors that may contribute to the effects of quarantine length on depression and anxiety in the general public under self-quarantine during the early phase of COVID-19 epidemic in China. It is expected that (a) quarantine length should predict depression and anxiety; (b) negative cognitive factors (including anticipation and worry) should mediate this relationship between them; (c) dispositional optimism, uncertainty tolerance, social support, and healthy behaviors should further moderate these relationships.

## Materials and Methods

### Participants and Procedures

Potential participants were recruited via online advertisements on social media. Using convenience sampling, 5,115 Chinese residents (72.75% females) were recruited in this online survey study between February 11, and February 19, 2020. The participants were from 32 provinces or autonomous regions including Hong Kong, Macao and Taiwan. Participants' age ranged from 15–71 years [mean = 21.27, standard deviation (*SD*) = 4.40]. In this sample, 0.59% of the participants (*n* = 30) were from Wuhan, 1.00% (*n* = 51) from other key regions designated by Shanghai, and the remaining 98.42% (*n* = 5,034) were from non-key regions^2^. Furthermore, 50.81% of the participants (*n* = 2,599) had family income lower than ¥50,000/year, 31.26% (*n* = 1,599) within ¥50,000/year - ¥100,000/year, 11.18% (*n* = 572) within ¥100,000/year–¥200,000/year, 3.09% (*n* = 158) within ¥200,000/year–¥300,000/year, and 3.66% (*n* = 187) higher than ¥300,000/year. For the level of education, 0.29% (*n* = 15) of the participants graduated from primary school, 0.04% (*n* = 2) from junior high school, 1.23% (*n* = 63) from senior high school, 2.07% (*n* = 106) from junior college, 92.84% (*n* = 4,749) from college, and 3.49% (*n* = 180) postgraduates.

The survey study was conducted online in computer and smartphone friendly format. Participation of the study was anonymous and voluntary. The average completion time was 8.98 min. No monetary compensation was provided to the participants. This study was approved by the body for ethical evaluation of research projects at the Department of Psychology—part of the School for Social and Behavioral Sciences at Nanjing University, China. All procedures contributing to this work comply with the ethical standards of the relevant national and institutional committees on human experimentation and with the Helsinki Declaration of 1975, as revised in 2008. Informed consent was obtained from all individual participants included in the study.

### Measures

#### Quarantine Length

The quarantine length was a self-report measure using a single survey question: “how many days have you been quarantined?” The score was as follows: 1 = 0 days, 2 = 1–7 days, 3 = 8–14 days, 4 = more than 15 days.

#### Depression

We used the depression subscale of Brief Symptom Inventory (Derogatis, 2000) to assess depression symptoms experienced in the past week. The scale comprises 6 items (e.g., “feeling blue” and “feeling hopeless about the future”). Items were rated on a 5-point scale, ranging from 0 (*not at all*) to 4 (*very much*), to indicate the extent to which each statement applied to the participant. The scale produced an acceptable Cronbach's alpha score of 0.84 in the current sample.

#### Anxiety

We used the anxiety subscale of Brief Symptom Inventory (Derogatis, 2000) to assess anxiety symptoms experienced in the past week. The scale comprises 6 items (e.g., “feeling tense” and “feeling suddenly scared”). Items were rated on a 5-point scale, ranging from 0 (*not at all*) to 4 (*very much*), to indicate the extent to which each statement applied to the participant. The scale had an acceptable Cronbach's alpha score of 0.90.

#### Worry

We used three items adapted from the McCaul Brief Worry Scale (McCaul and Goetz, n.d.) to assess worry (e.g., how worried are you about the coronavirus?). One item was rated on a 4-point scale, ranging from 1 (*never*) to 4 (*always*), and the other two items were rated on a 5-point scale , ranging from 1 (*not at all*) to 5 (*extremely*), to indicate the extent to which each statement applied to the participant. The scale had an acceptable Cronbach's alpha score of 0.81. The total score across the three items was used as a compound measure for worry.

#### Anticipation

We used two self-designed items to asses anticipation (i.e., “Do you think that you will contract coronavirus?” and “Do you think that your family will contract coronavirus?”). Items are rated on a 5-point scale, ranging from 1 (*Absolutely not*) to 5 (*Absolutely*), to indicate the extent to which each statement applies to the participant. The scale had an acceptable Cronbach's alpha score of 0.76.

#### Dispositional Optimism

We used the Life Orientation Test-Revised (Scheier et al., 1994) to assess dispositional optimism. The scale comprises 6 items (e.g., “In uncertain times, I usually expect the best” and “I hardly ever expect things to go my way”). Items were rated on a 5-point scale, ranging from 1 (*strongly disagree*) to 5 (*strongly agree*), to indicate the extent to which each statement applied to the participant. The scale had an acceptable Cronbach's alpha score of 0.72.

#### Tolerance of Uncertainty

We used the Intolerance of Uncertainty–Short Form (Carleton et al., 2007) to assess tolerance of uncertainty. The scale comprises 12 items (e.g., “Unforeseen events upset me greatly” and “It frustrated me not having all the information I need”). Items were rated on a 5-point scale, ranging from 1 (*not at all characteristic of me*) to 5 (*extremely characteristic of me*), to indicate the extent to which each statement applied to the participant. The scale had an acceptable Cronbach's alpha score of 0.87. We scored all items in reverse, so that the higher the score, the higher the tolerance of uncertainty.

#### Social Support

We used five items adapted from the Medical Outcomes Study Social Support Survey (Sherbourne and Stewart, 1991) to asses social support (e.g., Does your community often help your family?). Items were rated on a 4-point scale, ranging from 1 (*not at all*) to 4 (*extremely*), to indicate the extent to which each statement applied to the participant. The scale had an acceptable Cronbach's alpha score of 0.74.

#### Healthy Behavior

We used three self-designed items to assess how many days they engaged in three healthy behaviors over the past week (i.e., engaging in aerobic physical activity for at least 15 min, engaging in strengthening exercises, and eating fruits or vegetables). Items were rated on an 8-point scale, ranging from 1 (0 day) to 8 (7 days), to indicate the extent to which each statement applied to the participant. The scale had an acceptable Cronbach's alpha score of 0.65.

### Data Analysis

The Statistical Package for Social Sciences (SPSS) Version 22.0 and AMOS Version 22.0 were used for data analyses. Firstly, descriptive statistical analysis and statistical analysis of the differences in demographic variables were performed using independent *t*-test for binary factors (i.e., gender and key vs. non-key regions) or ANOVAs for multi-level factors (i.e., family income and education level). For these analyses, some groups with the number of participants fewer than 30 were combined to yield more robust estimates of the group means. For instance, for the current residence, Wuhan and other key regions were combined into one group as the key region. For the education level, we have combined primary school, junior high school, senior high school and junior college as the below-undergraduate group. Secondly, *Pearson* correlation analysis was used to explore the relationships between the main variables. On the basis of these correlation analyses, structural equation model (SEM) was subsequently used to assess the relationships between quarantine length, depression, anxiety, and the mediating effect of anticipation and worry. Finally, in order to investigate the moderated role of dispositional optimism, tolerance of uncertainty, healthy behavior and social support, SEM multiple-group analysis was carried out with these measures as grouping variables, respectively. In addition, according to the suggestions of Wen et al. (2004), root mean square error of approximation (RMSEA) lower than 0.08, and comparative fit index (CFI), normative fit index (NFI), and Tucker-Lewis index (TLI) higher than 0.90 are used as the cutoff criteria for goodness of fit indices in SEM. It should be noted that, given the large sample size, we define statistical significance for our purposes as effects at *p* < 0.01, as suggested by Sweeny et al. (2020). Furthermore, due to the low reliability of the health behavior scale, we deleted it in the later analysis and only investigated the moderated role of dispositional optimism, tolerance of uncertainty, and social support.

## Results

### Preliminary Analysis

The results of the descriptive statistic of each demographic variable were shown in Table 1. The results of independent sample *t*-tests showed that female participants scored significantly higher in anxiety, worry, dispositional optimism, tolerance of uncertainty, and social support than male participants. In addition, quarantine length and score of anxiety, worry, and anticipation were significantly higher in the key regions than those of non-key regions. An ANOVA yielded significant differences in all scales, except for depression, across the level of family annual income. Overall, quarantine length, worry, and anticipation, and tolerance of uncertainty of individuals with family annual income of more than ¥200,000 were worse than those with a family annual income of < ¥200,000. However, this high-income group also showed better experience in dispositional optimism and social support at the same time. In addition, education level had significant effects on all scales except tolerance of uncertainty. Specifically, quarantine length, depression, anxiety, worry, anticipation, dispositional optimism, and social support were significantly worse in postgraduates than those of the other two groups.

**Table 1:** Descriptive statistics of and difference in demographic variables of all study variables (*M* ± *SD*).

	**QL**	**Depression**	**Anxiety**	**Worry**	**Anticipation**	**DO**	**TU**	**SS**
Total	1.69 ± 1.20	3.42 ± 3.67	3.57 ± 4.02	6.78 ± 1.92	3.25 ± 1.42	22.77 ± 3.94	45.33 ± 7.97	13.23 ± 3.15
**Gender**								
Male	1.68 ± 1.18	3.44 ± 3.88	3.32 ± 4.18	6.45 ± 2.13	3.18 ± 1.45	21.92 ± 4.13	43.72 ± 8.57	12.75 ± 3.41
Female	1.69 ± 1.20	3.41 ± 3.59	3.66 ± 3.95	6.90 ± 1.82	3.27 ± 1.41	23.09 ± 3.82	45.93 ± 7.65	13.41 ± 3.02
*t*	−0.43	0.26	−2.65^**^	−6.91^***^	−2.02	−9.20^***^	−8.45^***^	−6.35^***^
*Cohen's d*	—	—	−0.08	−0.23	—	−0.29	−0.27	−0.20
**Current residence**								
Key regions	2.60 ± 1.46	4.21 ± 3.88	5.27 ± 4.94	7.59 ± 1.96	4.17 ± 1.63	22.01 ± 3.79	45.73 ± 7.51	13.22 ± 3.34
Non-key regions	1.68 ± 1.19	3.41 ± 3.66	3.54 ± 3.99	6.76 ± 1.92	3.23 ± 1.41	22.79 ± 3.94	45.32 ± 7.98	13.23 ± 3.14
*t*	5.69^***^	1.95	3.14^**^	3.85^***^	5.92^***^	−1.82	0.45	−0.03
*Cohen's d*	0.69	—	0.39	0.43	0.62	—	—	—
**Family income**								
< ¥50,000 (1)	1.62 ± 1.16	3.36 ± 3.63	3.50 ± 3.91	6.68 ± 1.89	3.16 ± 1.37	22.54 ± 3.97	45.59 ± 8.03	12.91 ± 3.19
¥50,000–¥100,000 (2)	1.67 ± 1.18	3.38 ± 3.50	3.41 ± 3.80	6.79 ± 1.83	3.25 ± 1.40	23.20 ± 3.77	45.42 ± 7.61	13.51 ± 2.99
¥100,000–¥200,000 (3)	1.83 ± 1.26	3.62 ± 3.86	3.91 ± 4.47	6.98 ± 2.03	3.38 ± 1.48	22.49 ± 4.04	43.91 ± 8.10	13.63 ± 3.17
¥200,000–¥300,000 (4)	2.09 ± 1.37	3.68 ± 4.06	4.23 ± 4.38	7.31 ± 1.98	3.85 ± 1.64	22.96 ± 4.11	45.12 ± 8.37	13.87 ± 3.10
>¥300,000 (5)	1.99 ± 1.32	3.7 ± 4.51	4.26 ± 5.10	6.90 ± 2.53	3.64 ± 1.74	23.07 ± 4.22	45.43 ± 9.04	13.63 ± 3.37
*F*	11.65^***^	1.23	4.28^**^	6.38^***^	14.73^***^	7.93^***^	5.31^***^	14.75^***^
*$\eta_{p}^{2}$*	0.01	—	0.003	0.01	0.01	0.01	0.004	0.01
*Post-hoc* tests (Bonferroni)	3 > 1; 4 > 1; 5 > 1; 4 > 2; 5 > 2	—	Ns	3 > 1; 4 > 1; 4 > 2;	3 > 1; 4 > 1; 4 > 2; 4 > 3; 5 > 1; 5 > 2	2 > 1; 2 > 3	1 > 3; 2 > 3	2 > 1; 3 > 1; 4 > 1; 5 > 1
**Education level**								
Below college degree (1)	1.76 ± 1.21	4.00 ± 4.21	4.26 ± 4.64	6.48 ± 2.10	3.37 ± 1.49	22.25 ± 4.18	44.84 ± 8.60	12.62 ± 3.25
Undergraduate (2)	1.67 ± 1.18	3.34 ± 3.58	3.44 ± 3.87	6.76 ± 1.89	3.20 ± 1.39	22.83 ± 3.93	45.34 ± 7.94	13.27 ± 3.15
postgraduate (3)	2.25 ± 1.41	4.79 ± 4.83	6.24 ± 5.70	7.52 ± 2.31	4.36 ± 1.61	21.80 ± 3.91	45.66 ± 8.04	12.63 ± 2.93
*F*	21.18^***^	15.99^***^	45.78^***^	15.73^***^	59.77^***^	7.62^***^	0.50	7.33^**^
*$\eta_{p}^{2}$*	0.01	0.01	0.02	0.01	0.02	0.003	—	0.003
*Post-hoc* tests (Bonferroni)	3 > 1; 3 > 2	3 > 2	1 > 2; 3 > 1; 3 > 2	3 > 1; 3 > 2	3 > 1; 3 > 2	2 > 3	—	2 > 3

*** *p < 0.001,*

** *p < 0.01.*

*QL, quarantine length; DO, dispositional optimism; TU, tolerance of uncertainty; SS, social support. The following are the same*.

### Correlation Analysis

As shown in Table 2, correlation coefficients among all variables were significant expect for the relationship between quarantine length and social support, between worry and social support, and between tolerance of uncertainty and social support.

**Table 2:** Intercorrelations between all study measures.

**Variables**	**1**	**2**	**3**	**4**	**5**	**6**	**7**	**8**
1. QL	1							
2. Depression	0.14^***^	1						
3. Anxiety	0.12^***^	0.73^***^	1					
4. Worry	0.13^***^	0.25^***^	0.35^***^	1				
5. Anticipation	0.13^***^	0.26^***^	0.29^***^	0.26^***^	1			
6. DO	−0.07^***^	−0.36^***^	−0.29^***^	−0.09^***^	−0.23^***^	1		
7. TU	−0.08^***^	−0.31^***^	−0.31^***^	−0.24^***^	−0.14^***^	0.29^***^	1	
8. SS	0.02	−0.20^***^	−0.14^***^	0.03	−0.07^***^	0.38^***^	0.03	1

*** *p < 0.001,*

**p < 0.01.

### Testing the Mediation Role of Worry and Anticipation

According to the results of the correlation analyses, quarantine length, worry, anticipation, depression, and anxiety were related to each other, which meets the requirements of the multiple mediation model (Marsh et al., 2004). SEM was thus used to further explore the mediation role of worry and anticipation with quarantine length as the independent variable and depression and anxiety as the dependent variable. It should be noted that:

a) As all variables are on single-dimension scales, they were treated as latent variables and their items were treated as explicit variables to better fit models.
b) Given the significant correlation between worry and anticipation, the correlation between worry and anticipation was established in order to avoid Type I error in expanding the model calculation results. More importantly, considering the often cooccurring and correlated depression and anxiety, we also established a correlation between depression and anxiety.
c) Because of the significant differences in several measures across gender, current residence, family income, and education level, these variables with significant differences were used as covariates to test the model among quarantine length, worry, anticipation, depression, and anxiety. However, for the sake of visual presentation of the results, they were not shown in the figures presented in this paper.

**Figure 1:**
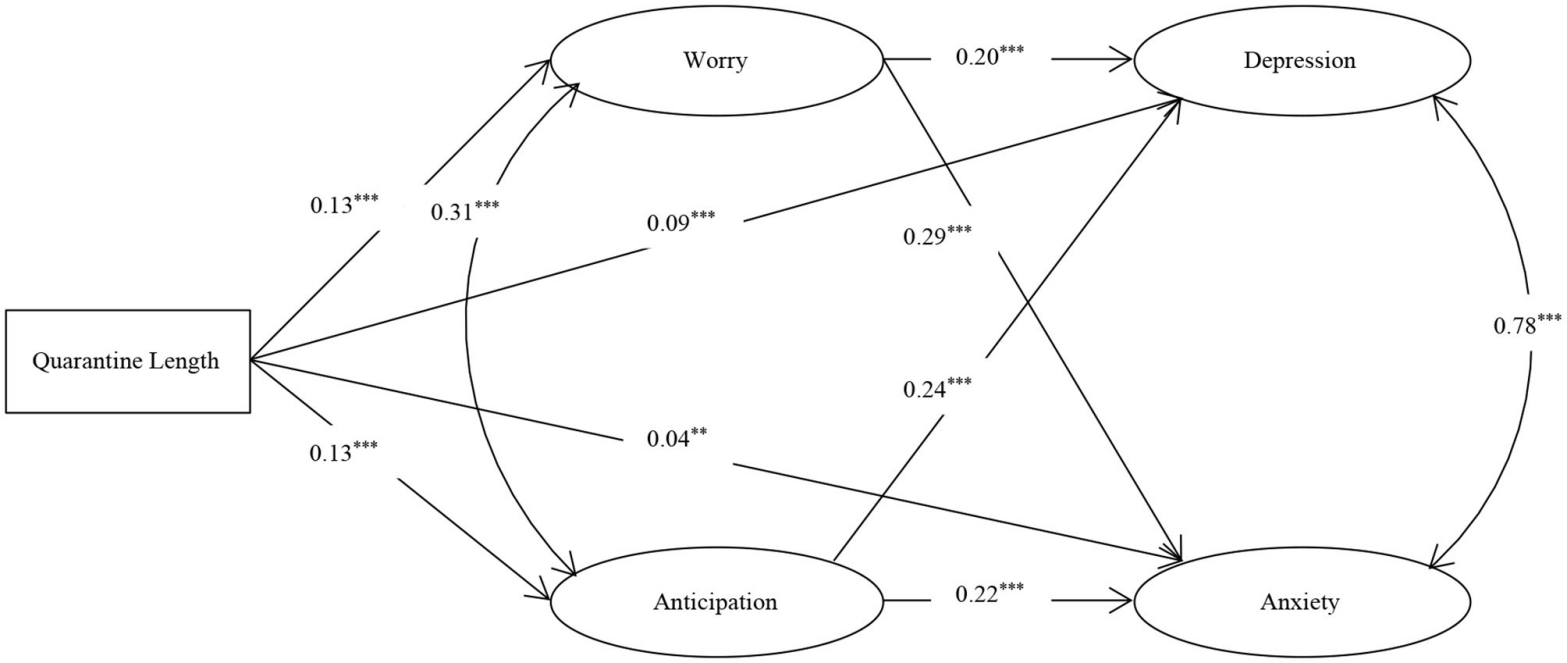
The multiple mediation model among quarantine length, worry, anticipation, depression, and anxiety. ***p < 0.001, **p < 0.01.

The SEM produced reasonable fits of the data (χ^**2**^*/df* = 5.19, RMSEA = 0.03, IFI = 0.98, CFI = 0.98, TLI = 0.98). As shown in Figure 1, further analyses of the paths in the model yielded significant predictive effects of quarantine length on depression (β = 0.09, *t* = 6.09, *p* < 0.001), anxiety (β = 0.04, *t* = 3.09, *p* = 0.002), worry (β = 0.13, *t* = 8.79, *p* < 0.001) and anticipation (β = 0.13, *t* =8.30, *p* < 0.001), significant predictive effects of worry on depression (β = 0.20, *t* = 11.58, *p* < 0.001) and anxiety (β = 0.29, *t* = 17.89, *p* < 0.001), and significant predictive effects of anticipation on depression (β = 0.24, *t* = 13.44, *p* < 0.001) and anxiety (β = 0.22, *t* = 13.57, *p* < 0.001). These results show that worry and anticipation contribute in a mediating role between quarantine length and depression. The mediating effect value was 0.06, accounting for 40.00% of the total effect^3^. Worry and anticipation also play a mediating role between quarantine length and anxiety. The mediating effect value was 0.07, accounting for 63.64% of the total effect.

### Testing the Moderated Role of Dispositional Optimism, Tolerance of Uncertainty, and Social Support

In order to investigate the moderated role of dispositional optimism, tolerance of uncertainty, and social support, SEM multiple-group analysis was carried out with these measures as grouping variables, respectively. Specifically, using the ±1 SD as the grouping standard, the high and low group were selected to fit the model in Figure 1, to analyze whether the model has significant effects across different levels of dispositional optimism, tolerance of uncertainty, and social support. According to the requirements of multiple-group comparison of models (Wen et al., 2003), the following three nested models were defined:

Model 1(unconstrained model): The same model structure was defined for different groups, without any restrictions on each parameter in the model;

Model 2 (measurement model): Based on Model 1, the path coefficients of different groups of measurement models were limited to be equal;

Model 3 (structural model): Based on Model 2, the path coefficients of different groups of structural model parts were limited to be equal.

If there was a significant difference between the two groups in some path coefficients, a simple slope test (Preacher et al., 2006) was conducted to further test the moderated effect of each moderated variable on the significant paths.

#### The Moderated Effect of Dispositional Optimism

**Figure 2:**
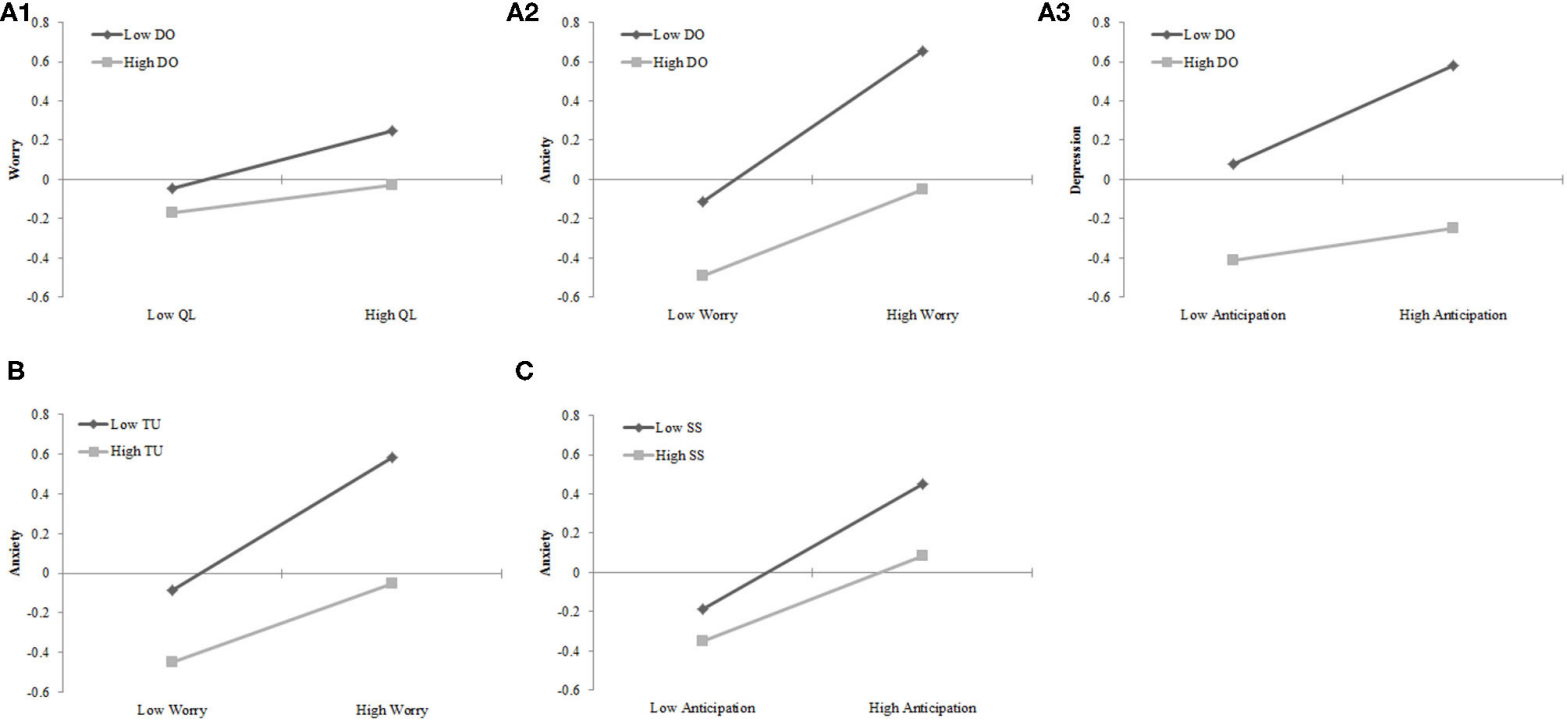
The moderated role of dispositional optimism, tolerance of uncertainty, and social support. **(A1)** The moderated effect of dispositional optimism on the relationship between quarantine length and worry. **(A2)** The moderated effect of dispositional optimism on the relationship between worry and anxiety. **(A3)** The moderated effect of dispositional optimism on the relationship between anticipation and depression. **(B)** The moderated effect of tolerance of uncertainty on the relationship between worry and anxiety. **(C)** The moderated effect of social support on the relationship between anticipation and anxiety.

The results showed that the model fit indices for Model 1, Model 2, and Model 3 were good (see Table 3). Further analyses showed significant differences between the high and low *Dispositional Optimism* groups in the measurement (Δχ^2^/Δ*df* = 19.51, *p* < 0.001) and structural models (Δχ^2^/Δ*df* = 14.63, *p* < 0.001). The difference between the measurement structural model was also significant (Δχ^2^/Δ*df* = 6.70, *p* < 0.001). The results of pairwise parameter comparisons showed that the path coefficients of quarantine length to worry (see Figure 2A1 and Table 4), worry to anxiety (see Figure 2A2 and Table 4), and anticipation to depression (see Figure 2A3 and Table 4) were significantly different between the two groups (*p* < 0.01).

**Table 3:** Models' fit indices.

**Model**	****χ^2^****	***df***	****χ^2^/*df*****	**CFI**	**NFI**	**TLI**	**RMSEA**
**Moderated variable: dispositional optimism**							
Model 1	706.10	338	2.09	0.98	0.96	0.97	0.02
Model 2	959.69	351	2.73	0.96	0.94	0.95	0.03
Model 3	1013.27	359	2.82	0.96	0.94	0.95	0.03
**Moderated variable: tolerance of uncertainty**							
Model 1	632.92	338	1.87	0.98	0.95	0.97	0.02
Model 2	697.29	351	1.99	0.97	0.95	0.97	0.03
Model 3	722.73	359	2.01	0.97	0.95	0.96	0.03
**Moderated variable: social support**							
Model 1	788.156	338	2.33	0.98	0.96	0.97	0.03
Model 2	858.25	351	2.45	0.97	0.95	0.96	0.03
Model 3	876.59	359	2.44	0.97	0.5	0.96	0.03

**Table 4:** Simple slope test results.

**Path**	**Moderated variable: DO**				**Moderated variable: TU**				**Moderated variable: SS**			
	**Low group (*****n*** **=** **914)**		**High group (*****n*** **=** **938)**		**Low group (*****n*** **=** **837)**		**High group (*****n*** **=** **705)**		**Low group (*****n*** **=** **1,065)**		**High group (*****n*** **=** **836)**	
	**β**	***t***	**β**	***t***	**β**	***t***	**β**	***t***	**β**	***t***	**β**	***t***
QL → Worry	0.15	7.72^***^	0.07	3.64^***^	—	—	—	—	—	—	—	—
QL → Anticipation	—	—	—	—	—	—	—	—	—	—	—	—
Worry → Depression	—	—	—	—	—	—	—	—	—	—	—	—
Worry → Anxiety	0.38	23.44^***^	0.22	12.40^***^	0.33	20.44^***^	0.21	10.89^***^	—	—	—	—
Anticipation → Depression	0.25	15.06^***^	0.09	4.25^***^	—	—	—	—	—	—	—	—
Anticipation → Anxiety	—	—	—	—	—	—	—	—	0.32	17.57^***^	0.22	11.01^***^
QL → Depression	—	—	—	—	—	—	—	—	—	—	—	—
QL → Anxiety	—	—	—	—	—	—	—	—	—	—	—	—

*Low group means those whose scores lower than M – 1 SD, while high group means those whose scores higher than M + 1SD.*

*** *p < 0.001,*

**p < 0.01.

#### The Moderated Effect of Tolerance of Uncertainty

The results showed that the model fit indices of Model 1, Model 2, and Model 3 were good (see Table 3). Further analyses showed significant differences between the high and low *Tolerance of Uncertainty* groups in the measurement (Δχ^2^/Δ*df* = 4.95, *p* < 0.001) and structural models (Δχ^2^/Δ*df* = 4.28, *p* < 0.001). The difference between the measurement and structural model was also significant (Δχ^2^/Δ*df* = 3.18, *p* < 0.001). The results of pairwise parameter comparison showed that the path coefficients of worry to anxiety (see Figure 2B; Table 4) was significantly different between the two groups (*p* < 0.01).

#### The Moderated Effect of Social Support

The results showed that the model fit indices of Model 1, Model 2, and Model 3 were good (see Table 3). Further analyses showed that there were significant differences between the high and low *Social Support* groups in the measurement (Δχ^2^/Δ*df* = 5.39, *p* < 0.001) and structural models (Δχ^2^/Δ*df* = 4.21, *p* < 0.001). The difference between the measurement and structural model was also significant (Δχ^2^/Δ*df* = 2.29, *p* = 0.02). The results of pairwise parameter comparison showed that the path coefficients of anticipation to anxiety (see Figure 2C and Table 4) was significantly different between the two groups (*p* < 0.01).

## Discussion

In this study, SEM was used to investigate the relationship between quarantine length and negative affect, as well as the roles of negative cognitions and several protective factors. The results showed that anticipation and worry partially mediated the relationship between quarantine length and negative affect. Dispositional optimism, tolerance of uncertainty, and social support moderated one or more paths of the relationship among quarantine length, anticipation, worry, depression, and anxiety. High dispositional optimism, high tolerance of uncertainty, and good social support can decrease the prediction effects of some paths.

### Demographic Factors

Consistent with longer quarantine in the key regions, anxiety, worry, and anticipation of infection were higher in residents from the key regions than those in residents from non-key areas. Interestingly, women experienced worse anxiety, and worry of infection than men did on the one hand, but better dispositional optimism, social support, and tolerance of uncertainty on the other hand, directly replicating a previous finding of increased experience in both negative and positive affect (Yue et al., 2017). These observations also replicated recent reports of gender differences in emotional disorders during the COVID-19 epidemic (Wang et al., 2020a,b). They are also consistent with the overall findings of higher rate of depression and anxiety in women (Altemus, 2006; Altemus et al., 2014) and higher rate of posttraumatic stress disorder (PTSD) after traumatic events in women (Breslau, 2009; Luxton et al., 2010). The gender differences in social support, dispositional optimism, and tolerance of uncertainty may result from women's better ability to utilize social support for psychological well-being (Flaherty and Richman, 1989) and to perceive happiness in daily life (Bradburn, 1969). It is worth noting that, although most of our samples are well-educated females, which was similar to the previous studies (Li et al., 2020; Ustun, 2020), it is still possible that the contradictory findings that women reported higher levels of anxiety and worry as well as optimism and social support may partially reflect a tendency to respond in an acquiescent manner in the collectivist culture, as suggested in Rammstedt et al. (2017). Therefore, caution is still advised before drawing conclusions.

Another demographic factor, family annual income, also had similar impacts on most measures. Specifically, worry, anticipation, and tolerance of uncertainty of individuals with family annual income of more than ¥200,000 were worse than those with a family annual income of < ¥200,000. However, this high-income group also showed better experience in dispositional optimism and social support at the same time. These seemingly contradictory findings may result from the association between the level of annual family income and the source of the family income. On the one hand, some industries have high income but low stability (e.g., self-employed households), while others have low to medium income but high stability (e.g., civil servants). It is possible that individuals with annual household income higher than ¥200,000 had less stability in maintaining their income. Consequently, worry, anticipation and intolerance of uncertainty of individuals were in these participants, which is consistent with a recent finding that the level of mental health of individuals with unstable family income was low in the epidemic (Cao et al., 2020). On the other hand, these participants with higher family income may expect speedy recovery of their income in the near future, which subsequently leads to higher dispositional optimism.

It is worth noting that quarantine length, depression, anxiety, worry, anticipation, dispositional optimism and social support were worse in postgraduates than participants with junior college degree or below. The worse emotional well-being in postgraduates in the present study is consistent with the overall findings of the potential mental health issues in postgraduates (e.g., Hou et al., 2013). For instance, Evans et al. (2018) conducted a comprehensive survey of 2,279 people through social media and e-mail and found that postgraduates were six times more likely to suffer from depression than the general population. Cao et al. (2020)'s study indicated that delays in academic activities was positively associated with college students' anxiety symptom. As far as we know, this phenomenon may be more obvious in postgraduate students because the epidemic makes them unable to return to school and lab to continue their research work, most of which can't be solved online like learning work. These delays in academic activities will lead to their delay in graduation, and further affect their future availability of jobs and incomes.

### Quarantine Length and Negative Affect

One of the key finding of the present study is that quarantine length, anticipation, worry, depression, and anxiety are correlated with each other. Removal of the social environment during the quarantine can be an important source of psychological stress. In the literature, lots of research on quarantine are based on analog simulation of space environment. For example, participants in bed rest for 60 days exhibited fluctuations (high-low-high-low) in depression and anxiety over time (Qin et al., 2010). Similarly, high prevalence of psychological distress was also reported in quarantined respondents to a web-based survey during the onset of SARS (Hawryluck et al., 2004). Specifically, symptoms of PTSD and depression were reported in 28.9 and 31.2% of the respondents in this study, respectively. In addition, longer durations of quarantine were associated with increased prevalence of PTSD symptoms. Consistent with previous these previous findings, our study also found the predictive effect of quarantine length on negative affect.

### The Mediating Roles of Worry and Anticipation

On the basis of correlation analysis, we further use SEM to investigate the mediating effect of anticipation and worry on quarantine length and negative affect. Consistent with the hypothesis, worry and anticipation about the COVID-19 mediate the relationship between quarantine length and negative affect. Specifically, the longer the durations of quarantine are, the greater anticipation and worry of individuals and their families to contract the virus are, which further worsens depression and anxiety symptoms. This result is in line with Schulz and Lazarus (2012)'s cognitive mediation theory and also a previous finding that negative cognition such as worry can mediate the relationship between stress events and depression (Young and Dietrich, 2015). Together these findings suggest that quarantine, as an acute stress event, can activate people's negative cognitive sensitivity, and consequently aggravating depression and anxiety.

### The Moderated Roles of Protective Factors

The results of multiple-group analyses highlighted three factors. First, dispositional optimism moderated the path coefficients of quarantine length to worry, worry to anxiety, and anticipation to depression. These novel findings are consistent with some previous evidence that dispositional optimism can act as a buffer. For instance, dispositional optimism can alleviate the relationship between stress and mental health (Chang, 1998), negative life events and suicide intention (Hirsch et al., 2007). This is because, as a protective factor, dispositional optimism can promote positive and future-oriented evaluations of external events and their negative physiological and psychological consequences (Brissette et al., 2002). Individuals with high level of dispositional optimism may be more positive in considering negative and potentially traumatic living environment than those with low level of dispositional optimism (Miller et al., 1996). Therefore, in the context of quarantine during the epidemic, individuals with higher dispositional optimism may prioritize the positive effects of lockdown measures such as the public health benefits over its negative impacts and adjust their lifestyle in a timely manner. That is, dispositional optimism provides a protective mental mechanism to buffer the effects of worry and anticipation on mental health (e.g., limit the growth of anticipation and worry over the quarantine period).

Second, tolerance of uncertainty moderated the path coefficient of worry to anxiety. Tolerance of uncertainty has recently gained research interests in the health care context, given the various sources of uncertainties in clinical setting (Hillen et al., 2017), including whether a patient has or will develop a particular condition; how that condition will evolve; to what extent a particular treatment is beneficial; and whether a patient is receiving the right care, in the right place, at the right time, and from the right people. Similarly, in the context of the epidemic, people who are quarantined face many uncertainties: when can the epidemic be effectively controlled; whether I or my family will be infected by the virus; how to maintain steady income to support family. Consequently, individuals with higher tolerance of uncertainty will experience less negative affect for a given level of worry.

Third, social support moderated the path coefficient of anticipation to anxiety. At present, the relationship between social support and physical and mental health has reached a general consensus, but the mechanisms of social support are still controversial (Cohen and Wills, 1985). On the one hand the main effect model states that high social support is often accompanied by better mental health; on the other hand, the buffer model asserts that social support only plays a significant role in high stress situations such that it will protect individuals from the adverse effects of stress (Cohen and Wills, 1985). The results of correlation analyses in this study showed that social support was negatively correlated with depression, anxiety, and anticipation, supporting the main effect model of social support. Results of multiple-group analysis, in line with previous findings (Khatib et al., 2013; Raffaelli et al., 2013), showed that social support moderated the path coefficient of anticipation to anxiety, supporting the buffer model of social support. These findings suggest that in the context of the epidemic, social support (including emotional comfort and practical help) from relatives, friends, and the community can effectively reduce negative affect.

### Implications and Limitations

This study examined the effects of quarantine length on negative affect during COVID-19 and the potential mediating and moderating factors. The moderating effects have identified dispositional optimism, uncertainty tolerance, and social support as potential psychological buffers for coping with the negative affect experienced during COVID-19. These protective factors are supplementary to those reported in a recent study (Wang et al., 2020b) that highlighted beneficial contributions of high level of confidence in doctors, perceived survival likelihood and low risk of contracting COVID-19, satisfaction with health information, and personal precautionary measures. These findings are highly informative for the society to develop strategies for mitigating public health crises such as the COVID-19 pandemic. In addition, our findings highlight potentially important practices, including dispositional optimism, uncertainty tolerance, and social support that the individuals could adopt to better cope with the pandemic in other impacted countries.

Nonetheless, this study has some caveats. Firstly, with a cross-sectional design (i.e., quarantine length is measured across respondents) and the regression approach, the present findings do not provide any evidence for a causal relationship between quarantine length and negative affect. Second, with the development of epidemic situation over time, the observed relationships among the various factors and measures may be dynamic and different at different key timepoints. As a result, it is unclear whether our current findings can be generalized from the study period (20 days after the outbreak) to other time points of this epidemic. Thirdly, to facilitate participant recruitment and to ensure the data quality for this online survey study at a particularly stressful time, we tried to limit the study length to roughly 10 min. Consequently, most measures in the present study used short scales instead of the complete scales. Although these short scales have been well-established in the literature, future research needs to implement the full scales to get a more systematical assessment of the various outcome measures. Fourthly, the short quarantine and measurement of quarantine length as Likert-5 scale rather than accurate days limits the statistical power, which affected the effect sizes, especially the relationship involving quarantine length. Therefore, caution is still advised before drawing conclusions. Fifthly, the method of multiple-group analysis in moderated effect tests leads to waste of participants, so the results of the moderated variable as continuous variables as shown in the Supplements can also be considered. Sixthly, due to the number of non-key regions was significantly larger than the number of non-key regions (27 vs. 636 cities), 1.59% of participants were from the key regions, whereas approximately 98.41% of participants were from the non-key regions in this study, so the present findings may mainly reflect the epidemic related mental health issues in these non-key regions. Seventhly, the method of convenient sampling limits the generalization of our conclusion. On the one hand, according to study demographics, the samples essentially represent Chinese female college students, as indicated by gender ratio (72.75% females) and average age (21.27 ± 4.40); on the other hand, based on the significant negative correlations between completion time and quarantine length (*r* = −0.04, *p* = 0.004) and depression (*r* = −0.06, *p* < 0.001), and significant positive correlations between completion time and dispositional optimism (*r* = −0.21, *p* < 0.001), tolerance of uncertainty (*r* = −0.13, *p* < 0.001), and social support (*r* = −0.06, *p* < 0.001), survey completers represent the subset of the distribution most interested in survey content although these effect sizes are relatively low. Take these two aspects together, these should be very cautious when conclusions are generalized to other populations. Eighthly, we focused on the situation of voluntary quarantine at home in the current study, future research should further examine the negative affect in forced quarantine and the differences between forced quarantine and voluntary quarantine. Finally, although the similar increased levels of depression and anxiety during epidemics have been demonstrated in many countries (e.g., US; Ettman et al., 2020), and the worry about COVID-19 infection in Japan was similar to the results of this study (Sasaki et al., 2020), it is still necessary to conduct further cross-cultural studies to compare these variables (e.g., intensity, frequency, and interpretation of worry) and their relationship in the future.

## Conclusion

This study found that quarantine length could predict depression and anxiety. This relationship is further mediated by worry and anticipation about the COVID-19, and moderated by several protective factors, including dispositional optimism, uncertainty tolerance, and social support.

## Data Availability Statement

The raw data supporting the conclusions of this article will be made available by the authors, without undue reservation.

## Ethics Statement

The studies involving human participants were reviewed and approved by the body for ethical evaluation of research projects at the Department of Psychology—part of the School for Social and Behavioral Sciences at Nanjing University, China. The patients/participants provided their written informed consent to participate in this study.

## Author Contributions

LH, FL, and YM collected the data. LH and RZ analyzed and interpreted the data. LH, XC, and WZ wrote the current version of the manuscript. All authors have made a significant contribution to this work.

## Conflict of Interest

The authors declare that the research was conducted in the absence of any commercial or financial relationships that could be construed as a potential conflict of interest.

## Footnotes

^1^According to Centers for Disease Control and Prevention (https://www.cdc.gov/quarantine/), quarantine is the separation and restriction of movement of people who have potentially been exposed to a contagion disease to assure if they become unwell, so reducing the risk of them affecting others. The quarantine in this study specifically refers to self-quarantine at home, which was a voluntary practice recommended by the government when the pandemic was started in China.

^2^We adopted the classification launched by the Shanghai Government, China. Key regions refer to the regions with more confirmed cases and were perceived to have a higher level of risk of infection. Non-key regions refer to the regions with no or fewer confirmed cases and were perceived to have a lower level of risk of infection. According to the classification of Shanghai City, 27 cities in China were classified as key regions, whereas all other cities in China were classified as non-key regions. Different home quarantine policies were applied to individuals returning from key vs. non-key regions. For instance, people in the key regions need to be quarantined at for a longer period of time than those who were in the non-key regions (14 vs. 7 days).

^3^The mediating effect value was calculated by the sum of the path coefficient from the independent variable to the mediated variable multiplied by the path coefficient from the mediated variable to the dependent variable. And the proportion of the mediating effect was calculated by the sum of the mediating effect divided by the mediating effect plus the direct effect.
