# Supplementary material for: The Relationship Between Quarantine Length and Negative Affect During the COVID-19 Epidemic Among the General Population in China: The Roles of Negative Cognition and Protective Factors
Source: Front Psychol. 2021 Apr 28;12:575684. doi: 10.3389/fpsyg.2021.575684 (PMC8113411; doi:10.3389/fpsyg.2021.575684)
Supplement: Supplementary file 1 [file Data_Sheet_1.docx]

**Supplements**

**Part 1. Main variables’ details**

**
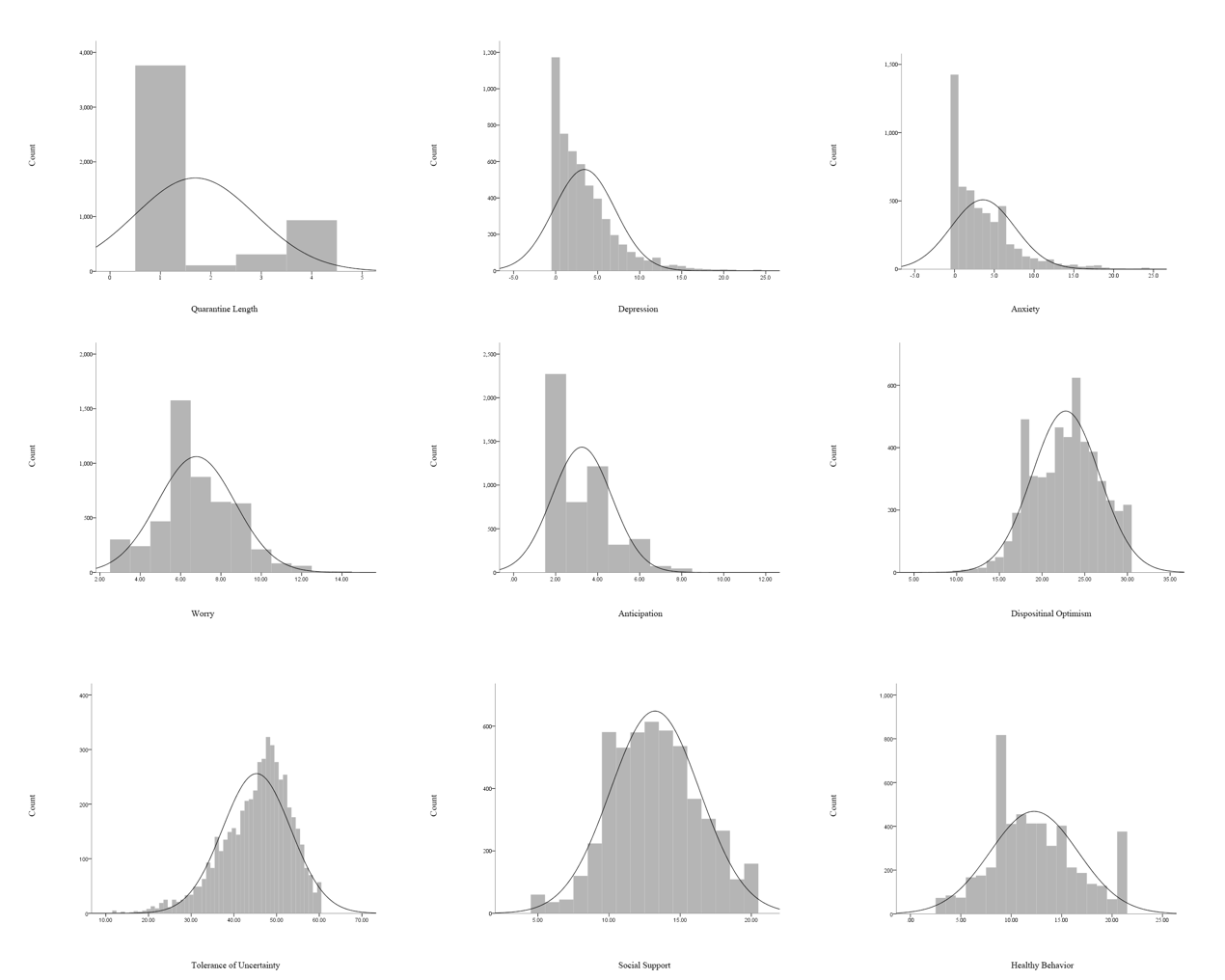
**

**Figure 1.** The histogram of quarantine length, depression, anxiety, worry, anticipation, dispositional optimism, tolerance of uncertainty, social support, and healthy behavior, respectively.

**
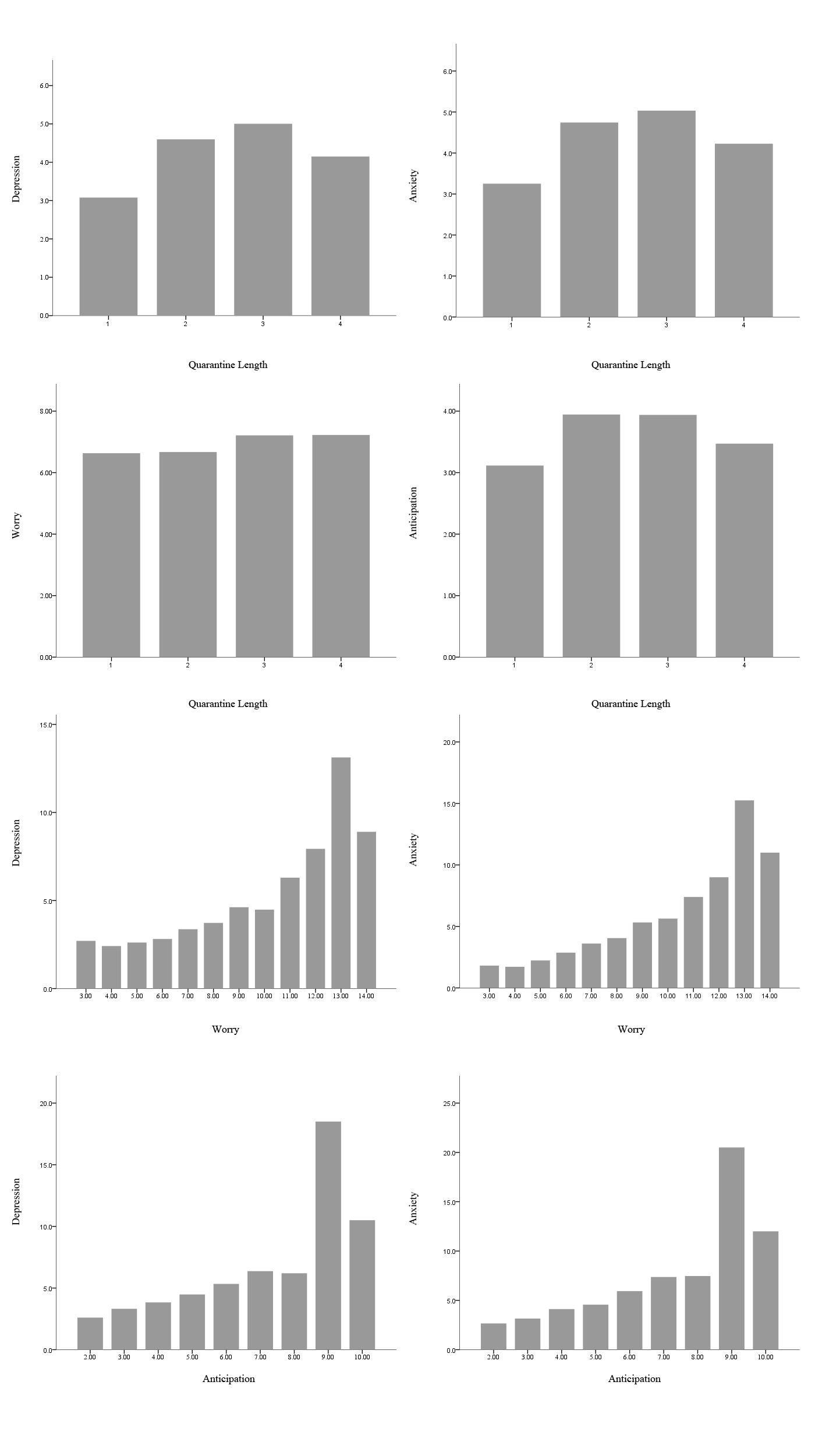
**

**Figure 2.** The relationships among quarantine length, worry, anticipation, depression, and anxiety.

**Part2. The results for moderator as continuous variables**

**Table 1.** Models’ fit indices

| Moderated variable | χ**^2^** | *df* | χ**^2^**/*df* | CFI | NFI | TLI | RMSEA |
| --- | --- | --- | --- | --- | --- | --- | --- |
| Dispositional Optimism | 1451.90 | 233 | 6.23 | 0.97 | 0.97 | 0.96 | 0.03 |
| Tolerance of Uncertainty | 1343.57 | 234 | 5.74 | 0.98 | 0.97 | 0.97 | 0.03 |
| Social Support | 1126.65 | 233 | 4.84 | 0.98 | 0.98 | 0.97 | 0.03 |

**Table 2.** The moderate effect results for moderator as continuous variables

| Path | Moderator | | |  |
| --- | --- | --- | --- | --- |
|  | Dispositional Optimism | Tolerance of Uncertainty | Social Support | |
| QL → Worry | 0.12^***^ | 0.11^***^ | 0.13^***^ | |
| QL → Anticipation | 0.11^***^ | 0.12^***^ | 0.13^***^ | |
| QL → Depression | 0.08^***^ | 0.08^***^ | 0.10^***^ | |
| QL → Anxiety | 0.03 | 0.03^**^ | 0.05^***^ | |
| Anticipation → Depression | 0.14^***^ | 0.22^***^ | 0.22^***^ | |
| Anticipation → Anxiety | 0.15^***^ | 0.20^***^ | 0.21^***^ | |
| Worry → Depression | 0.19^***^ | 0.14^***^ | 0.21^***^ | |
| Worry → Anxiety | 0.27^***^ | 0.23^***^ | 0.29^***^ | |
| Moderator → Worry | -0.12^***^ | -0.27^***^ | 0.01 | |
| Moderator→ Anticipation | -0.26^***^ | -0.16^***^ | -0.09^***^ | |
| Moderator → Depression | -0.23^***^ | -0.26^***^ | -0.21^***^ | |
| Moderator → Anxiety | -0.34^***^ | -0.22^***^ | -0.14^***^ | |
| QL*Moderator → Worry | -0.04^**^ | -0.03 | 0.001 | |
| QL*Moderator → Anticipation | -0.04^**^ | -0.04 | 0.02 | |
| QL*Moderator → Depression | -0.02 | 0.01 | -0.02 | |
| QL*Moderator → Anxiety | 0.001 | 0.01 | -0.02 | |
| Anticipation*Moderator → Depression | -0.08^***^ | -0.01 | -0.05^***^ | |
| Anticipation*Moderator → Anxiety | -0.07^***^ | -0.01 | -0.05^***^ | |
| Worry*Moderator → Depression | -0.04^**^ | -0.03 | 0.002 | |
| Worry *Moderator→ Anxiety | -0.05^***^ | -0.06^**^ | 0.01 | |

Note: ^***^ *p* < .001, ^**^ *p* < .01. The font marked in red is the difference between the two test methods.
